# Implementation challenges in preeclampsia care: perspectives from health care professionals in urban Uganda

**DOI:** 10.1016/j.xagr.2024.100348

**Published:** 2024-04-02

**Authors:** Imelda Namagembe, Babu Karavadra, Lawrence Kazibwe, Joseph Rujumba, Noah Kiwanuka, Brandon Smith, Josaphat Byamugisha, Ashley Moffett, Tom Bashford, Annettee Nakimuli, Catherine E. Aiken

**Affiliations:** a Department of Obstetrics and Gynaecology, Makerere University, Mulago National Referral Hospital (Dr Namagembe, Dr Kazibwe, and Drs Rujumba, Byamugisha, and Nakimuli), Kampala, Uganda; b Norwich Medical School, University of East Anglia (Dr Karavadra), Norwich, United Kingdom; c Department of Epidemiology and Biostatistics, School of Public Health, Makerere University (Dr Kiwanuka), Kampala, Uganda; d International Health Systems group, Department of Engineering, University of Cambridge (Drs Smith and Bashford), Cambridge, United Kingdom; e Department of Pathology and Centre for Trophoblast Research, University of Cambridge (Dr Moffett), Cambridge, United Kingdom; f Department of Obstetrics and Gynaecology, University of Cambridge, Rosie Hospital, NIHR Cambridge Biomedical Research Centre (Dr Aiken), Cambridge, United Kingdom

**Keywords:** focus groups, health care, obstetrics, maternal health, morbidity, mortality, multidisciplinary, preeclampsia, quality improvement, resources, thematic analysis, Uganda

## Abstract

**BACKGROUND:**

Sub-Saharan Africa bears the burden of 70% of maternal deaths worldwide, of which ∼10% are attributable to hypertensive disorders of pregnancy, primarily complications of preeclampsia. In other global settings, outcomes of pregnancies affected by preeclampsia are improved with timely and effective medical care.

**OBJECTIVE:**

This study aimed to explore the perspectives of local health care professionals on how preeclampsia care is currently delivered in the study setting and what challenges they experience in providing prompt and safe care. We identified specific objectives of exploring stakeholder perceptions of (1) recognizing preeclampsia and (2) timely intervention when preeclampsia is diagnosed. We also explored the wider system factors (eg, cultural, financial, and logistic challenges) that health care professionals perceived as affecting their ability to deliver optimal preeclampsia care.

**STUDY DESIGN:**

Individual semistructured interviews were conducted with health care professionals and stakeholders. The findings were analyzed using thematic analysis.

**RESULTS:**

Thirty-three participants contributed to the study, including doctors and midwives with varying degrees of clinical experience and external stakeholders. The following 5 key themes emerged: delayed patient presentation, recognizing the unwell patient with preeclampsia, the challenges of the existing triage system, stakeholder disconnect, and ways of learning from each other. Health care professionals referenced an important psychosocial perspective associated with preeclampsia in the study setting, which may influence the likelihood of seeking care through traditional healers rather than hospital-based routes.

**CONCLUSION:**

We identify the key barriers to improving maternal and neonatal outcomes of preeclampsia, described at both the institutional level and within the wider setting. The study provides invaluable contextual information that suggests that a systems-based approach to health care quality improvement may be effective in reducing rates of maternal and neonatal morbidity and mortality.

**AJOG MFM at a Glance**

**Why was this study conducted?**

Preeclampsia is a life-threatening obstetrical condition for which prompt and appropriate care can improve maternal and neonatal outcomes.

**Key findings**

The following 5 key themes emerged from the study: delayed presentation for care, recognizing the unwell patient with preeclampsia, the challenges of robust triage, stakeholder disconnect, and ways of learning from each other.

**What does this add to what is known?**

We identify the key barriers to improving maternal and neonatal outcomes in preeclampsia, described at both the institutional level and within the wider setting. The study provides valuable contextual information that suggests that a systems-based approach to quality improvement may be effective in reducing rates of maternal and neonatal morbidity and mortality.

## Introduction

Sub-Saharan Africa bears the burden of 70% of maternal deaths worldwide, of which approximately 10% are attributable to hypertensive disorders of pregnancy, primarily complications of preeclampsia.^1^ Compared with women in other global settings, African women are more likely to experience preeclampsia, which affects up to 10% of all pregnancies.^2^ In urban Uganda, preeclampsia contributes to 25% of maternal deaths^3^ and is a key determinant of fetal growth and survival.^4^

In other settings, prompt and effective recognition and treatment of preeclampsia significantly improves maternal and neonatal outcomes.^5^ However, the time-sensitive nature of preventing complications of preeclampsia presents a significant challenge, especially in very high-acuity maternity settings. We have previously demonstrated that timely acute obstetrical interventions are difficult to achieve within the study setting,^6^ which is one of the busiest maternity units in Africa, delivering >25,000 infants annually. A wide range of strategies have been adopted elsewhere to address the difficulties of achieving timely intervention in preeclampsia. These include, for example, early-warning systems,^7^ new clinical management guidelines,^8^ and educational interventions.^9^ However, it is currently unclear which strategies would be most effective when integrated into a systems approach to preventing maternal deaths from preeclampsia in urban Uganda.

We aimed to explore the perspectives of local health care professionals on how preeclampsia care is currently delivered in the study setting and what challenges they experience in providing prompt and safe care.

## Materials and Methods

### Reflexivity statement

The study team consisted of an international partnership of clinical academics with expertise in maternal health in both Uganda and the United Kingdom. The study objectives were informed by their personal and clinical experiences and the wider experience of maternal care in Uganda. The research questions were framed within a biopsychosocial paradigm, and thus the study design is informed by a context-specific understanding of Uganda from both emic and etic perspectives.

### Recruitment and sampling

The study was conducted in a national maternity referral center in Kampala, Uganda, as part of a wider project examining maternal mortality in urban Uganda (August 2021 to January 2022).^10^ Participants were encouraged to participate in the study through word of mouth and additional advertisement in the referral center. Purposive sampling occurred initially, followed by snowball sampling. All participants were provided with an information leaflet about the study and were given up to 2 weeks to consider their participation.

Eligible participants were health care professionals working in the study center or representatives of the Ugandan Ministry of Health. Participants were informed that their responses would not identify them personally and they could withdraw from the study until fully anonymized data were collated.

The data were collected by a Ugandan clinician (I.N.), and the findings were transcribed verbatim. Consent was obtained in person at the time of interview by the research team. All interviews were audio-recorded. Participant personal data were linked to research data using an anonymized alphanumeric key and were stored independently of primary research data.

The study was approved by the Makerere University School of Medicine Research and Ethics Committee (SOMREC; 2018-001) and by the Uganda National Council for Science and Technology (UNCST; SS4797). The data collection process was continued until data saturation^11^ was reached at 33 interviews. Saturation was determined by reflective conversations among the research team during data collection when new interviews failed to generate new codes.

### Interview methodology

A topic guide was used to conduct the interviews.^10^ Information on participant demographics (age, occupation) was collected, and open-ended questions were posed regarding the participants’ perceptions of factors affecting preeclampsia care. A phenomenological perspective was taken, based on analyzing respondents’ subjective experiences to make generalizable statements about the recognition and timely management of preeclampsia in Uganda. The interview guide was trialed with a Ugandan obstetrician with extensive experience of working in the study context to ensure participant acceptability. Individual interviews (lasting 30–60 minutes) were conducted to enable participants to voice their thoughts freely without the effect of perceived power dynamics between different stakeholder groups.

### Data analysis

The findings were anonymously collated using NVivo 9 (2010; Lumivero, Denver, CO) for thematic coding, and analyzed using phenomenologically oriented reflexive thematic analysis.^12^ This analysis structure constituted the following: (1) familiarization with the data set, (2) generating initial codes, (3) identifying themes within the codes, (4) refining the themes, and (5) defining the themes. This structure was applied as an interactive and iterative process moving between the different stages.

## Results

Themes were derived from analysis of the entire hermeneutic unit of 33 interviews. Participant characteristics are described in the Table. The identified themes (Figure) are presented as a narrative describing the clinical pathway of preeclampsia care and the wider factors identified as influencing the trajectory of care.

**Table:** Participant demographics

Job title	Age range	Mean age	Sex		Total
			Female	Male	
Specialist doctors in obstetrics and gynecology (referred to as Consultants)	34–56	41.7	3	7	10
Midwives	32–55	42.2	6	0	6
Junior doctors	29–44	33.2	3	4	7
Ministry of Health representatives	35–54	43.6	2	8	10
Total	29–56	40.9	14	19	33

**Figure:**
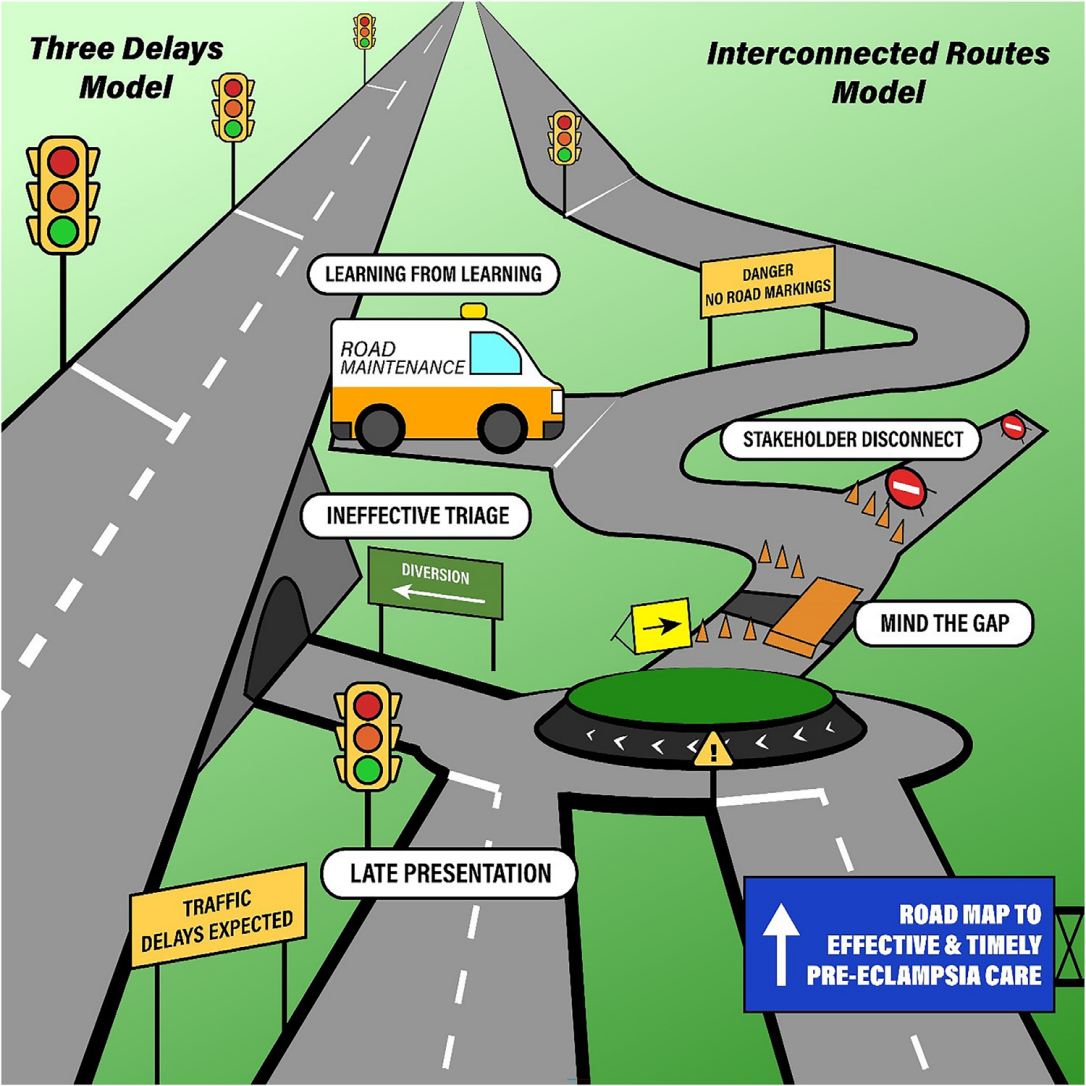
Major themes identified from reflexive thematic analysis of study data

Namagembe. Preeclampsia care in urban Uganda. Am J Obstet Gynecol Glob Rep 2024.

### Theme 1: challenges of late presentation

Several participants commented that women may not recognize that their pregnancy is medically complex and therefore may not appreciate that they are at increased risk of hypertensive disease in pregnancy.

“The patient comes from a household. The other members of the household may take a long time to identify that this is a complicated pregnancy.” (Consultant #1)

Women with possible preeclampsia may not seek medical attention immediately when they recognize that they are unwell, but instead consult with a non–health care professional first.

“Sometimes they go to the nearest health facility where the attendant is not skilled, and that may delay recognizing a complicated pregnancy.” (Consultant #1)

Midwives explained that the signs and symptoms of preeclampsia may be misinterpreted by patients.

“Patients don't even see their symptoms as preeclampsia and then get help in the community with the healer before coming to the hospital.” (Midwife #1)

Addressing the idea that women did not recognize preeclampsia symptoms, several health care professionals explained that good antenatal care can increase awareness of pregnancy-associated complications and would therefore encourage women to attend the hospital for assessment.

“We need to educate mothers on why they should attend clinics to avoid complications.” (Midwife #2)

Addressing the wider contextual factors associated with late presentation for care, participants explained that there were significant difficulties with the local transport system, and expressed their frustration with the challenges that women experienced in reaching the hospital.

“The ambulance system in Kampala is weak. Some are traveling on boda-bodas, others in taxis, others in special-care cars.” (Consultant #2)

Health care professionals expressed the opinion that late presentation for care was mainly due to factors outside of their influence, but emphasized the impact of these challenges on their ability to provide safe care.

### Theme 2: ineffective triage system

All clinicians identified the existing triage system as a barrier to providing timely assessment and care for women with suspected or confirmed preeclampsia.

The concept of an effective triage system was discussed in relation to ensuring that all women presenting to the hospital have a blood pressure reading done upon arrival. The participants identified efficient blood pressure measurement as a key step to enabling rapid preeclampsia treatment. This would also facilitate a timely medical review and reduce the delay to diagnosis.

“BP should be taken in triage on arrival, and if raised, a doctor can see them straight away.” (Midwife #3)

However, although some staff felt confident to commence early therapy in the acute setting, others described a lack of awareness and uncertainty around current clinical guidelines. This was cited, particularly by midwives who are usually responsible for triage, as a barrier to recognizing and therefore promptly managing preeclampsia in the hospital setting.

“Most people do not know how to manage preeclampsia, as they rely on outdated knowledge.” (Midwife #4)

The lack of qualified staff available to manage emergency presentations and the high turnover of patients were frequently mentioned as factors affecting the assessment of patients with suspected or confirmed preeclampsia.

### “The manpower should be enough to handle high numbers of patients.” (Doctor)

“The turnover of the patients is high and staffing is low.” (Doctor #1)

Several participants noted that daily uncertainty about available supplies can impact rapid clinical decision-making.

“Sometimes, supplies are in short stock. Sometimes, decisions take a long time to be made.” (Midwife #1)

Several participants commented on how supplies were organized and available in the emergency setting. There was recognition that teamwork is critical in treating severe preeclampsia. The role of the “emergency team” was discussed to facilitate safe management for those with preeclampsia.

“Emergency teams are needed. But, when you don't have an emergency trolley, you can lose life. People need to have a specific role in this emergency too.” (Midwife #5)

The importance of ensuring that the most appropriately qualified health care professional reviews each patient was widely discussed in the interviews. There was no mention of how critically unwell patients are escalated between health care professionals, whether from a midwife to a doctor, or between doctors with differing levels of experience. However, there was recognition that immediate medical decisions made at triage by recently qualified health care professionals (for example, interns and residents) should later be reviewed by those with more experience.

“A regular senior doctor ward round should happen. If the juniors are not supported, then it can lead to near-misses or deaths.” (Doctor #2)

### Theme 3: recognizing the gap between identifying an unwell patient and commencing treatment

Some doctors explained that “mild preeclampsia” is not always viewed seriously by clinicians and that these patients are overlooked following initial admission to the hospital. Several experienced doctors discussed that health care professionals risk underestimating the speed of progression from mild to severe preeclampsia, especially when there are competing priorities on their time. Participants referred to an increasing awareness that mild preeclampsia can progress and lead to maternal death and felt that there should be an ongoing review pathway for these women.

“Last year we identified that most deaths were from mothers with mild PET. These women were on the ward for so long, and so we started to eventually see even the mild PET patients on ward round, which we didn't before.” (Doctor #3)

Clinical pressures in managing the workload and the vast number of patients presenting to the hospital also contributed to the challenges in recognizing and managing preeclampsia. The available skill mix within the multidisciplinary health care team was also recognized as an important factor in providing safe care.

“The high numbers of patients is a risk, but the skill mix of the health care professionals also needs to be looked at.” (Doctor #3)

“Too much work and then failing to prioritize.” (Junior Doctor #4)

### Theme 4: stakeholder disconnect

Both clinicians and managerial stakeholders agreed that ensuring that clinical areas are well resourced is a crucial aspect of improving patient care. However, different stakeholder groups had varying perspectives on the pathways to achieving this.

“The ED and the hospital administrator need to understand that most solutions are not costly.” (Junior Doctor #1)

“If we could have independent-minded leaders who can make decisions for the good of the patients, then we will achieve better.” (Midwife #1)

“The government is committed to having facilities equipped.” (Government stakeholder #1)

However, there was awareness among all stakeholders, including nonclinicians, that preeclampsia is life-threatening to the mother and the neonate, and can lead to maternal death if not managed appropriately.

In terms of coordinating patient care with external stakeholder institutions, such as lower-level health care facilities, participants commented how they did not always have a clear understanding of the referral pathways between various institutions. Health care professionals reported that it was crucial that patients be cared for in the appropriate medical facility to meet their care needs, but identified the lack of a well-connected referral network as detrimental to achieving this.

“We need national referral guidelines in place so that you know what each facility can offer.” (Doctor #4)

“We need to have proper guidelines and we need to disseminate them.” (Midwife #5)

Staff members in more direct patient-facing roles tended to discuss specific logistical problems, often citing examples related to particular equipment or medications. By contrast, those in managerial roles and those whose work was further removed from clinical practice tended to discuss the competing demands of resourcing services overall.

### Theme 5: learning from each other

The final element that participants strongly agreed on was the need for a reflective clinical service that can learn from cases with poor outcomes. An iterative approach to refining care for women with critical illness, including severe preeclampsia, to improve outcomes was considered critical. The importance of a nonblame culture was discussed by participants as essential to allow this approach to be developed.

“We need a nonblame environment, and only then will we be able to change things.” (Midwife #5)

Clinicians explained that after a significant event, such as a maternal death in the context of preeclampsia, any recommendations made should not be generic, but specific to the precise problem to inform realizable improvements in care.

“Recommendations are generated, but sometimes they are not specific enough to enable change.” (Doctor #3)

The importance of discussing any lessons learned from significant medical events was mentioned by several participants. In particular, the way in which learning was disseminated was considered important.

“After identifying the gaps, the recommendations need to be actually disseminated to everyone so we can learn from it.” (Junior Doctor #1)

## Discussion

### Principal findings

Several themes were identified in relation to the barriers in providing effective care for women with preeclampsia. Health care professionals discussed the psychosocial perspective associated with preeclampsia in Uganda and the influence of this on health-seeking behaviors.

### Clinical implications

The “three delays” model^13^ has been used to understand pregnancy-related mortality as a function of delays in the following: (1) seeking help, (2) reaching an appropriate facility, and (3) receiving adequate care. However, this linear model does not fully explain how care may not be appropriate and timely within complex health care systems. We identified barriers to prompt and effective care for women with preeclampsia in urban Uganda. Health care professionals tended to focus on elements that were present within the clinical setting, such as issues with triage, but there was also a widespread appreciation that preeclampsia care is influenced by the cultural context in which it occurs. Participants discussed factors such as community recognition of pregnancy complications, resourcing of health care, and transport infrastructure in Kampala as influencing the clinical outcomes of the population.

Participants recognized the importance of having an effective triage system for rapidly identifying patients with preeclampsia. Our previous studies suggest that delays in effective care after reaching the hospital contribute to 64% of all maternal deaths in the study context, and that >80% of maternal deaths from hypertensive disorders are considered preventable by local clinician review panels.^3^ Participants identified barriers related to organization of resources, acuity on the clinical frontline, and an appropriate mix of skills and experience within the health care team on shift. The concept of a dedicated emergency team with specific allocated resources was referenced in interviews with several participants as needing further development within the clinical context.

Prompt initial triage was considered crucial, particularly because many maternal deaths occur shortly after arrival at the study center.^3^^,^^14^ However, we also identified issues with postemergency care. Participants referenced the value of experienced clinical input beyond the initial presentation of preeclampsia, and spoke of the risk to women whose condition at presentation was not severe. Deterioration in preeclampsia is unpredictable,^15^ and participants perceived that strengthened systems for ongoing review could result in better outcomes. This challenges the three delays model given that a woman may seek care at an appropriate facility in a timely manner, and yet receive care that is not sufficient for the severity of her underlying condition. Our focus may need to shift toward strengthening systems to deliver quality care.^16^

Participants also spoke about detailed cultural factors that influence complex health care–seeking behavior among local mothers. These included competing demands on resources and logistical issues, but also cultural expectations of health in pregnancy and previous health care–related experiences.^17^ Health care professionals referenced an important psychosocial perspective associated with preeclampsia in the study setting, which may influence the likelihood of seeking care through traditional healers rather than hospital-based routes. Research in other African contexts has revealed a significant mythology in relation to preeclampsia, including themes related to “witchcraft, ghost attacks, and stress from strained relationships.”^18^ Our results suggest that further qualitative research should be developed to explore the perception of preeclampsia in urban Ugandan communities.

Our findings suggest that a health care systems-based approach could be effectively applied to providing preeclampsia care in urban Uganda. A systems-based approach considers the wider context of providing health care in complex clinical environments.^19^ A striking finding within our study are the multiple levels at which participants referenced challenges for care provision, from community health engagement to high-level health care policy. A recent systematic review of approaches to health care quality improvement supported the application of systems-based methods to target effective interventions.^20^ This is particularly important in low-resource settings, where multiple levels of highly context-dependent issues must be overcome.^21^ Our work to understand the challenges of delivering preeclampsia care within the context of a very busy maternity unit in sub-Saharan Africa is an important first step toward applying a systems-based approach and refining care pathways for women with preeclampsia in this context (Figure).

### Research implications

Future work should involve a constructivist study of women and their caregivers across Uganda to understand the complex reasons for these systemic issues and how they differ within and between regions and subcultures.

### Strengths and limitations

Our study adopts a postpositivist stance to understand preeclampsia, grounded in an international scientific discourse. Respondents were interviewed in English, and all held technical or professional appointments. Despite this, key cultural and psychosocial issues were identified. Many of our respondents are likely to have personal experiences of childbirth, and we did not explore the tension between these and their professional experiences. The key strength of our sampling method is that it integrated the expert purposive knowledge of the research team with the broader perspectives provided by interviewees. However, we acknowledge that a limitation of this study is the potential for missing stakeholders who fall outside this scope. Examples of groups that could be explored further include health care professionals who refer patients to the study center, and a broader range of associated health care professionals within the study center. One potential limitation of our study design is that differing perspectives might have been expressed had the participants been engaged via other methodologies (for example, a survey-based study). However, the face-to-face interviews we conducted provide rich, detailed, and nuanced insights, which are a major strength of the study. Indeed, we did not explore the different ideas around preeclampsia care that might be articulated in different languages. Instead, we sought to make an objective contribution to understanding the current clinical systems around preeclampsia management in Kampala and extrapolate these to the broader Ugandan context.

### Conclusions

Hypertensive disorders of pregnancy contribute to approximately 1 in 4 maternal deaths in urban Uganda.^3^ Our results provide important insight into the challenges that stakeholders face in providing timely and effective preeclampsia care described at both the institutional level and within the wider setting. The study provides invaluable contextual information that suggests that a systems-based approach may be effective in reducing rates of maternal and neonatal morbidity and mortality.

## CRediT authorship contribution statement

**Imelda Namagembe:** Writing – review & editing, Writing – original draft, Formal analysis, Data curation. **Babu Karavadra:** Writing – review & editing, Writing – original draft, Formal analysis. **Lawrence Kazibwe:** Writing – review & editing, Formal analysis. **Joseph Rujumba:** Writing – review & editing. **Noah Kiwanuka:** Writing – review & editing, Formal analysis. **Brandon Smith:** Writing – review & editing, Formal analysis. **Josaphat Byamugisha:** Writing – review & editing, Formal analysis, Conceptualization. **Ashley Moffett:** Writing – review & editing, Formal analysis, Conceptualization. **Tom Bashford:** Writing – review & editing, Formal analysis. **Annettee Nakimuli:** Writing – review & editing, Formal analysis, Conceptualization. **Catherine E. Aiken:** Writing – review & editing, Writing – original draft, Formal analysis, Conceptualization.
